# Postoperative Cognitive Dysfunction after Coronary Artery Bypass Grafting

**DOI:** 10.21470/1678-9741-2018-0165

**Published:** 2019

**Authors:** Shi-Min Yuan, Hong Lin

**Affiliations:** 1 Department of Cardiothoracic Surgery, The First Hospital of Putian, Teaching Hospital, Fujian Medical University, Putian, Fujian Province, People's Republic of China.; 2 Department of Cardiology, The First Hospital of Putian, Teaching Hospital, Fujian Medical University, Putian, Fujian Province, People's Republic of China.

**Keywords:** Cognitive Dysfunction, Coronary Artery Bypass, Coronary Artery Bypass, Off-Pump, Postoperative Complications

## Abstract

Postoperative cognitive dysfunction is a common complication following cardiac surgery. The incidence of cognitive dysfunction is more pronounced in patients receiving a cardiac operation than in those undergoing a non-cardiac operation. Clinical observations demonstrated that pulsatile flow was superior to nonpulsatile flow, and membrane oxygenator was superior to bubble oxygenator in terms of postoperative cognitive status. Nevertheless, cognitive assessments in patients receiving an on-pump and off-pump coronary artery bypass surgery have yielded inconsistent results. The exact mechanisms of postoperative cognitive dysfunction following coronary artery bypass grafting remain uncertain. The dual effects, neuroprotective and neurotoxic, of anesthetics should be thoroughly investigated. The diagnosis should be based on a comprehensive cognitive evaluation with neuropsychiatric tests, cerebral biomarker inspections, and electroencephalographic examination. The management strategies for cognitive dysfunction can be preventive or therapeutic. The preventive strategies of modifying surgical facilities and techniques can be effective for preventing the development of postoperative cognitive dysfunction. Investigational therapies may offer novel strategies of treatments. Anesthetic preconditioning might be helpful for the improvement of this dysfunction.

**Table:** 

Abbreviations, acronyms & symbols	
βAP	= β-amyloid peptide
CABG	= Coronary artery bypass grafting
CAM	= Confusion Assessment Method
CFQ	= Cognitive Failures Questionnaire
CPB	= Cardiopulmonary bypass
isoP	= iso-prostane
MoCA	= Montreal Cognitive Assessment
MMSE	= Mental Status Examination
NfH	= Neuro-filament heavy chain
NSE	= Neuron-specific enolase
POCD	= Postoperative cognitive dysfunction

## INTRODUCTION

Postoperative cognitive dysfunction (POCD), characterized by impairment of attention, concentration, and memory with possible long-term implications, is a frequent neurological sequela following cardiac surgery. According to duration, POCD can be classified into two types: short-term and long-term. The former is usually a transitory cognitive decline lasting up to 6 weeks after a cardiac operation with an incidence of 20-50%, whereas the latter can be a subtle deterioration of cognitive function occurring six months after an operation with an incidence of 10-30%^[1]^. However, POCD might occur several years after an operation. The incidence of POCD depends on the types of operation, and it is more pronounced in patients receiving a cardiac operation than in those undergoing a non-cardiac operation^[2]^. A retrospective study demonstrated that coronary artery bypass grafting (CABG) is the most common cause of POCD after a cardiac operation with an incidence of 37.6% in 7 days and 20.8% in the 3^rd^ month of the postoperative period^[3]^.

POCD should be distinguished with relevant concepts, such as postoperative delirium and vascular dementia. Postoperative delirium is an acute mental syndrome characterized by a transient fluctuating disturbance of consciousness, attention, cognition, and perception as a common complication of surgery, occurring in 36.86% of the surgical patients^[4]^. The significant risk factors responsible for postoperative delirium were peripheral arterial disease, preexisting cerebrovascular disorders, and a reduced preoperative Hasegawa-dementia score^[5]^. POCD is a subtler deficit affecting patients' cognition, including verbal, visual, language, visuospatial, attention, and concentration aspects^[4]^. A more delayed development of POCD may indicate a poorer prognosis. Associated POCD and delirium was noted in 77% of aortic surgical patients at discharge, which suggests that both conditions could share a common mechanism^[4]^. Vascular dementia, as a result of any vasculopathies, is the most common type of dementia in elderly patients, who often have significant impairment of social or occupational functions, usually accompanied by focal motor and sensory abnormalities^[6]^.

Cognitive assessments between conventional CABG with the use of cardiopulmonary bypass (CPB) (on-pump) and CABG without CPB (off-pump) yielded inconsistent results. The exact mechanisms of development of POCD following CABG and cognitive impact of anesthetics remain uncertain. The diagnosis and treatment of POCD are still challenging. This article aims to present an overview of POCD following CABG.

### Predictive Risk Factors

Predictive risk factors of POCD may include old age, preexisting cerebral, cardiac, and vascular diseases, alcohol abuse, low educational level, and intra- and postoperative complications^[7]^. A univariant analysis revealed that older age, female gender, higher bleeding episodes, and increased postoperative creatinine levels were more significantly associated with POCD^[8]^. Laalou et al.^[9]^ described that POCD was age- and observational time-related, with an incidence of 23-29% in patients aged 60-69 and >70 years in one week, and 14% in those aged >70 years in the 3^rd^ month of the postoperative period. Cerebral hypoperfusion is an important risk factor contributing to postoperative brain damage, especially in atherosclerotic patients due to hypoperfusion-induced impaired clearance of microemboli and worsened ischemic damage^[10]^. POCD can result from systemic or cerebral inflammation due to neuronal injuries^[11]^. Surgical operation might trigger brain mast cell degranulation, microglia activation, and release of inflammatory cytokines, thus, leading to neuronal damages, and activated brain mast cells might induce neuronal apoptosis^[12]^. The association between increased levels of plasma inflammatory mediators (interleukin-1, interleukin-6, tumor necrosis factor-α, and C-reactive protein) and cognitive dysfunction has been found in postoperative patients and could eventually predict future cognitive decline^[13]^. Autonomic nervous suppression in relation to neuroendocrine response as well as cytokine production to surgery and anesthesia might play an important role in the development of POCD^[9]^. Other conditions like increased plasma cortisol levels via stimulation of the hypothalamic-pituitary axis^[9]^ and preexisting cerebrovascular disease^[14]^ could be specific risk factors of POCD.

### Diagnosis

#### Neuropsychiatric Tests

POCD is verified by employing batteries of psychometric tests performed pre- and postoperatively to assess cognitive performance. Currently, there is no gold standard, but some tests (Rey auditory verbal learning test, Trail-making A, Trail-making B, and Grooved pegboard) were recommended as core tests as proposed by Murkin et al.^[15]^ in 1995. Typically, a battery of tests is composed of a comprehensive assessment of the cognitive status, including memory, attention, language, executive function, and motor speed^[14]^. The Mini-Mental Status Examination (MMSE), a commonly used screening test for dementia with remarkable validity and reliability^[16]^, is sometimes used to quantify POCD^[17]^, and an MMSE value below 25 is regarded as POCD^[18]^. However, comparing cognitive function before operation and adjustment of age or education level is important before determining the POCD. In addition, the Confusion Assessment Method (CAM) and the Cognitive Failures Questionnaire (CFQ) were used pre- and postoperatively to evaluate the cognitive status^[18]^. The neuropsychiatric tests that are used in clinical practice are shown in Table 1^[8,15,16,18-21]^. Habib et al.^[8]^ noted that the McNair scale was more evident than MMSE. Proust-Lima et al.^[21]^ found that MMSE and Benton Visual Retention Test showed a superior sensitivity over Isaacs Set Test and Digit Symbol Substitution Test.

**Table 1:** Neuropsychiatric tests for cognitive assessment in clinical practice.

Year	Author	Neuropsychiatric tests
1995	Murkin et al.^[15]^	Core tests (Rey Auditory Verbal Learning Test, Trail Making A, Trail Making B, and Grooved Pegboard)
2002	Stroobant et al.^[19]^	Rey Auditory Verbal Learning Test (AVLT) (verbal memory), Trail Making Test (TMT Part B) (speed for visual search, attention and mental flexibility), Grooved Pegboard Test (GPT) (finger and hand dexterity), Block Taps Test (TAPS) (non-verbal immediate memory and attention), Line Bisection Test (LBT) (unilateral visual inattention), Controlled Oral Word Association Test (COWAT) (word fluency), and Judgement of Line Orientation (JLO) (ability for angular relationships between line segments)
2010	Benabarre et al.^[20]^	Positive and Negative Symptom Scale, Global Assessment Functioning, Wechsler Adult Intelligence Scale (WAIS), Wisconsin Card Sorting Test (WCST), Stroop Test, Trail Making Test (TMT), California Verbal Learning Test (CVLT), Wechsler Memory Scale (WMS), and Phonetic Verbal Fluency/Controlled Oral Word Association Tests
2007	Proust-Lima et al.^[21]^	Benton Visual Retention Test
2012	Jildenstål et al.^[18]^	Confusion Assessment Method (CAM) and Cognitive Failures Questionnaire (CFQ)
2014	Habib et al.^[8]^	McNair Scale
2015	Saraçlı et al.^[16]^	Mini-Mental Status Examination (MMSE)

#### Cerebral Biomarker Inspections

Biomarkers in relation to POCD have been described as β-amyloid peptide (βAP), *tau*, *phospho-tau*, apoE, interleukin-6, C-reactive protein, cortisol, S100β, and neuron-specific enolase (NSE). Increased serum *tau* level was seen in patients with cognitive decline after CPB, and increased cerebrospinal fluid βAP and *tau* levels were similar to those having Alzheimer's disease. Serum interleukin-6, C-reactive protein, and NSE are important indicators of POCD following CABG^[22]^. NSE, S100, and S100β are more sensitive for detecting cerebral structural and functional damages in patients undergoing various cardiac operations, and all peaked at the end of CPB^[23]^. Alternative potential indicators for POCD include neuro-filament heavy chain (NfH), iso-prostane (isoP), and reduced cerebral perfusion/hypoxia^[22]^.

#### Electroencephalography

Increased lower frequencies, reduced complex activities, and incoherent cortical regions/fast rhythms shown on the electroencephalogram may indicate POCD^[22]^.

### Literature Review

**Fig. 1:**
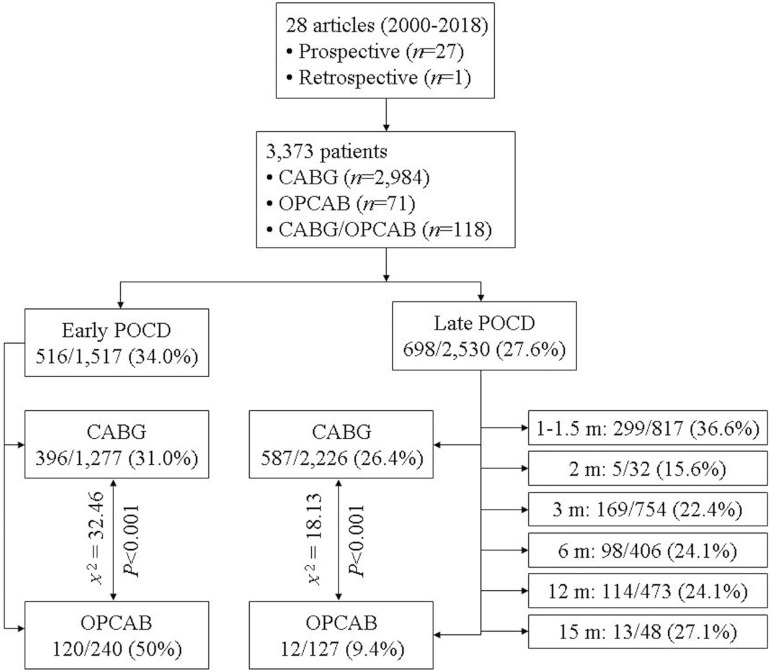
The outcomes of the review with a depiction of early and late postoperative cognitive dysfunctions following coronary artery bypass grafting. *Comparisons of frequencies were made by Fisher's exact test. CABG=coronary artery bypass grafting; m=months; OPCAB=off-pump coronary artery bypass; POCD=postoperative cognitive dysfunction

Pertinent literature was retrieved for articles published between 2000-2018. The search terms included "coronary artery bypass grafting", "on-pump", "off-pump", "cognitive decline", "postoperative cognitive dysfunction", "diagnosis", and "treatment". A total of 28 prospective or retrospective research articles were obtained which involved 3.373 patients^[24-51]^. The early and late rates of POCD were 34% and 27.6%, respectively. The statistical analysis made by Fisher's exact test for comparisons of frequencies showed that the early POCD rate was lower, but the late POCD rate was higher in CABG in comparison to OPCAB patients (Figure 1). The disparities in the early and late POCD incidences compared to the results reported in the literature were probably due to the differences in literature selection. Additionally, the literature review outstood some attenuators and intensifiers of POCD following CABG procedures (Table 2).

**Table 2:** Literature review of representative publications on postoperative cognitive dysfunction following coronary artery bypass grafting.

Author, year	Type of study	Patient number (CABG/OPCAB)	Intervention (parameter)	POCD, n (%)	Recommendation	Outcome	
						Attenuator	Intensifier
Kumpaitiene et al.^[24]^, 2018	Prospective observational study	59/0	Erythromycin (rSO_2_, NSE and GFAP)	22 (37) on POD10	No significant changes in blood GFAP level occurred in any patients; decreased rSO_2_ and increased NSE level did not correlate with rate of POCD	Erythromycin	
Thomaidou et al.^[25]^, 2017	Prospective randomized pilot study	40/0	Erythromycin (25 mg/kg) *vs*. control (Serum IL-1, IL-6 and *tau*)	19 (47.4) *vs*. 16 (76.2) just after hospital discharge, and 6 (31.6) *vs*. 38 (95.2) in 3 months		Erythromycin	
Kok et al.^[26]^, 2017	Randomized clinical trial	57 (CABG or OPCAB)	(Serum brain fatty acid-binding protein)	15 (26) in 3 months, and 13 (27) in 15 months	Classical neuronal injury-related biomarkers had no prognostic value for POCD		
Silva et al.^[27]^, 2016	Prospective observational study	88/0	(Serum S100β and NSE)	23 (26.1) at 21 days, and 20 (22.7) in 6 months	Serum S100B was more accurate than NSE in the detection of POCD		
ÖztÜrk et al.^[28]^, 2016	Prospective, randomized, double-blind study	40/0	Pulsatile vs. nonpulsatile flow (Serum 100β and NSE)	3 (15) *vs*. 3 (15) on POD3	No difference between types of pump flow for POCD		
Oldham et al.^[29]^, 2015	Prospective observational cohort study	102/0		14 (14) on POD2 through discharge	Cognitive and functional impairment independently predicted postoperative delirium and delirium severity		Preoperative cognitive and functional impairment
Hassani et al.^[30]^, 2015	Randomized, double-blind, placebo-controlled trial	61/0	Valerian capsule (containing 530 mg of valerian root extract/capsule) (1,060 mg/day) *vs*. placebo capsule each 12 h for 8 weeks	Valerian prophylaxis: reduced odds of POCD in comparison to placebo on POD10 and in 2 months		Valerian capsule	
Dong et al.^[31]^, 2014	Prospective cohort study	108/0	(Plasma copeptin)	35 (32.4) on POD7	Postoperative plasma copeptin level may be a useful predictor of POCD		
Trubnikova et al.^[32]^, 2014	Case-control study	101/0	MCI* vs*. non-MCI	36 (72) *vs*. 40 (79) at early stage (*P*=0.5), and 36 (72) *vs*. 35 (70) in 1 year (*P*=0.8)	MCI was not a leading cause of early or long-term POCD		
Kok et al.^[33]^, 2014	Randomized pilot study	29/30	CABG *vs*. OPCAB (Cerebral oximetry variable)	11 (39) *vs*. 4 (14) at early stage (*P*=0.50), and 4 (14) *vs*. 0 (0) at 3 months (*P*=0.03)	There was no association between intraoperative cerebral oximetry variables and POCD at any stage	OPCAB	CABG
Szwed et al.^[34]^, 2014	Prospective observational single-surgeon trial	0/74	"No-touch" OPCAB *vs*. "traditional" OPCAB	10 (28.6) *vs*. 20 (51.3) on POD7 (discharge)		"No touch" OPCAB	
Fontes et al.^[35]^, 2014	Retrospective study	118/0 (CABG or CABG + valve surgery with CPB)	Arterial hyperoxia during CPB	53 (45) at 6 weeks	Arterial hyperoxia during CPB was not associated with neurocognitive decline after 6 weeks		
Sirvinskas et al.^[36]^, 2014	Prospective study	50/0	Head-cooling *vs*. no head-cooling	9 (36) *vs*. 16 (64) on POD10 (*P*=0.048)		Head-cooling technique during the aortic cross-clamp	
Joung et al.^[37]^, 2013	Randomized pilot study	0/70	rIPC *vs*. control	10 (28.6) *vs*. 11 (31.4) on POD7 (*P*=0.794)	rIPC did not reduce the incidence of POCD		
Mu et al.^[38]^, 2013	Prospective cohort study	166/0	(Serum cortisol)	66 (39.8) on POD7			High serum cortisol level on POD1
Kadoi et al.^[39]^, 2011	Prospective study	124/0	Normal *vs*. medium *vs*. impaired cerebrovascular CO_2_ reactivity	20 (30) *vs*. 10 (25) *vs*. 11 (57) on POD7, and 16 (24) *vs*. 9 (23) *vs*. 5 (26) at 6 months			Impaired cerebrovascular CO_2_ reactivity
de Tournay-Jettéet al.^[40]^, 2011	Prospective study	61 (CABG or OPCAB)	(rSO_2_)	46 (80.7) on POD4-7, and 23 (38.3) in 1 month			Intraoperative rSO_2 _desaturation
Slater et al.^[41]^, 2009	Prospective controlled study	240/0	(rSO_2_ saturation)	70 (29) in 3 months	Patients with rSO_2_ desaturation score >3,000%-second had a significantly higher risk of POCD		rSO_2_ desaturation score >3,000%-second
Haljan et al.^[42]^, 2009	Prospective study	32/0	Erythropoietin *vs*. placebo	2 (8.3) *vs*. 3 (38) in 2 months (*P*=0.085)		Erythropoietin	
Silbert et al.^[43]^, 2008	Prospective study	282/0	(Apolipoprotein genotype)	33 (12) in 3 months, and 31 (11) in 12 months	There was no relationship between presence of the apolipoprotein epsilon4 allele or any of the six genotypes and POCD		
Jensen et al.^[44]^, 2008	Prospective randomized study	47/43	CABG *vs*. OPCAB	4 (9) *vs*. 8 (19) in 12 months (*P*=0.18)	No significant differences in the incidence of POCD between CABG and OPCAB group		
Hogue et al.^[45]^, 2008	Prospective study	113/0 (CABG/CABG+ valve operation)	(Apolipoprotein epsilon4 genotype)	28 (25) in 4-6 weeks	Mild atherosclerosis of the ascending aorta, CPB time, aortic cross-clamping time and length of hospitalization, but not apolipoprotein epsilon4 genotype were risks for POCD		
Puskas et al.^[46]^, 2007	Prospective study	525/0	Hyperglycemic *vs*. nonhyperglycemic	157 (40) *vs*. 38 (29) at 6 weeks (*P*=0.017)			Intraoperative hyperglycemia
Kadoi and Goto^[47]^, 2007	Prospective study	106/0	Sevoflurane *vs*. non-sevoflurane	13 (22) *vs*. 11 (23) in 6 months	Sevoflurane did not have any significant effects on POCD		
Kadoi and Goto^[48]^, 2006	Prospective study	88/0		24 (27.3) in 6 months	Age, diabetes mellitus and renal failure were associated with POCD at 6 months		
Jensen et al.^[49]^, 2006	Prospective randomized study	51/54	OPCAB *vs*. CABG	4 (7.4) *vs*. 5 (9.8) in 3 months (*P*=0.7)	No significant difference in the incidence of POCD between OPCAB and CABG		
Silbert et al.^[50]^, 2006	Prospective randomized study	326/0	High-dose fentanyl *vs*. low-dose fentanyl	22 (13.7) *vs*. 40 (23.6) in 1 week (*P*=0.03)		High-dose fentanyl	Low-dose fentanyl
Wang et al.^[51]^, 2002	Prospective randomized study	88/0	Lidocaine *vs*. placebo	8 (18.6) *vs*. 18 (40.0) at early stage (?) (*P*=0.028)		Intraoperative administration of lidocaine	

CABG=coronary artery bypass grafting; CO2=carbon dioxide; CPB=cardiopulmonary bypass; GFAP=glial fibrillary acidic protein; IL=interleukin; MCI=mild cognitive impairment; NSE=neuron-specific enolase; OPCAB=off-pump coronary artery bypass; POCD=postoperative cognitive dysfunction; POD=postoperative day; rIPC=remote ischemic preconditioning; rSO_2_=regional cerebral oxygen

Aykut et al.^[52]^ prospectively compared the effect of pulsatile and nonpulsatile flow on cognitive functions of patients undergoing CABG. The cognitive performance was evaluated with the Montreal Cognitive Assessment (MoCA) test one day before and one month after the operation. They observed an overall POCD rate of 17.3% in pulsatile and 35.6% in nonpulsatile flow group. Besides, mild cognitive impairment was seen more in the nonpulsatile than in the pulsatile flow group. This result was interpreted as pulsatile flow ensuring a lower systemic vascular resistance and higher oxygen consumption. The pulsatile flow might increase cerebral blood flow, aerobic metabolism, and oxygen delivery, and reduce cerebral vascular resistance^[53]^. On the contrary, the nonpulsatile flow does not possess these advantages^[54]^. Pulsatile perfusion preserves microcirculatory perfusion over the conventional nonpulsatile perfusion during CPB. The latter is associated with altered microvascular blood flow, increased leukocyte activation, endothelial dysfunction, and increased microvascular resistance along with elevated lactate as an indicator of tissue hypoxia^[55,56]^.

Some authors reported significant cognitive improvement in the first year follow-up period^[57]^, less cognitive impairment in one week^[58]^, less deteriorated cognitive scores in one and 10 weeks^[59]^, and better neuropsychological performance six months after surgery^[19]^ in OPCAB patients. Emmert et al.^[60]^ reported that on-pump CABG showed an increased risk of postoperative stroke. In contrast, the incidence of stroke was low in patients receiving a standardized OPCAB with no-touch techniques for a proximal anastomosis. They hypothesized that the underlying mechanisms of POCD were probably in virtue of cerebral mircroembolic, inflammatory, and non-physiological perfusion of CPB. However, reports revealed no difference in early postoperative cognitive impairment and late cognitive recovery between on-pump CABG and OPCAB procedures^[61-63]^. Such inconsistent results were explained by different patient selection criteria and low cardiac output during distal anastomoses in OPCAB^[62]^. Thus, van Dijk et al.^[64]^ concluded that POCD was insignificantly influenced by CPB. In line with this statement, Selnes et al.^[14]^ claimed that the late POCD was more likely resulted from preexisting cerebrovascular disorders other than from CPB. The possible explanation for this pervasive inconsistency was that different measures were taken concerning the use of unspecified diagnostic criteria for the assessment of POCD^[65]^, incongruousness of definition of cognitive decline, disparity of statistical methods, and lack of randomized control studies^[14]^.

### Management

#### Preventive Measures

Preoperative cognitive screening is a cost-effective way of preventing POCD^[14]^. Intraoperative monitoring of regional cerebral oxygen saturation against prolonged brain desaturation could be associated with an induced incidence of POCD^[66]^. The measures for preventing POCD by modifying surgical facilities, such as reducing particulate and gaseous microemboli (by using cardiotomy, cell-saver, arterial line filtration, anastomotic devices and cannulae with modified blood entry and less shear stress, and membrane oxygenator other than bubble oxygenator), hemostasis (by glucose management, temperature regulation and pH management) and surgical techniques, including OPCAB technique, minimized aortic manipulation with single other than multiple aortic clamping^[67,68]^, no-touch technique^[34]^, and pulsatile rather than non-pulsatile CPB^[69]^.

#### Therapeutic Strategies

Potential pharmacologic strategies include investigational (such as piracetam, cholinesterase inhibitors, glutamate N-methyl-D-aspartate antagonists, glutamate α-amino-3-hydroxy-5-methyl-4-isoxazolepropionic acid receptor modulators, γ-aminobutyric acid-B antagonists, nicotinic receptor agonists, dopamine/norepinephrine re-uptake inhibitors, *Ginkgo biloba*, coenzyme Q, antioxidants, and growth factors) and therapeutic agents with neuroprotective effects for immediate treatment of POCD (such as sedatives, acetylcholine sterase inhibitor, stimulants, statins, calcium channel antagonist and N-methyl-D-aspartate antagonist)^[69]^. However, these agents warrant further evaluations with regard to their long-term efficacies^[70]^. Moreover, investigations have proved that anesthetic preconditioning managements may improve POCD incidence. Royse et al.^[71]^ investigated the influence of propofol or desflurane on POCD incidence and found that desflurane was associated with a reduced incidence of early POCD in comparison to propofol (49.4% *vs*. 67.5%, *P*=0.018). This was interpreted as those anesthetics being potentially neurotoxic but sometimes neuroprotective for ischemia-reperfusion injury^[72]^.

The evaluation of anesthetics on cognitive outcome seems to be difficult. This is because anesthetics showed dual neurological impacts: neuroprotective and neurotoxic^[73]^. Mechanistic studies have been concentrated on ion channels of the nerve cells, particularly on receptors including α-amino-3-hydroxy-5-methyl-4-isoxazolepropionic acid, N-methyl-D-aspartic acid, γ-aminobutyric acid type A, glycine, 5-hydroxytryptamine type 3, and nicotinic acetylcholine receptors^[74]^. The neuroprotective effects of anesthetics may rely on the reduction of neuronal excitation and enhancement of γ-aminobutyric acid type A receptor function^[75]^. Anesthetic-induced cognitive impairment probably depends on the type and dose of anesthetics, the mode and route of drug delivery, and observational time^[2]^.

## CONCLUSION

The underlying etiologies of POCD following CABG can be complex. A comprehensive postoperative cognitive evaluation with neuropsychiatric tests, cerebral biomarker inspections, and electroencephalographic examination should be performed to assess patients' cognitive status. Inconsistent results of POCD between OPCAB and on-pump CABG warrant further evaluations in well-designed prospective studies. The preventive strategies of modifying surgical facilities and techniques can be effective for preventing the development of POCD. Investigational therapies may offer novel strategies of treatments for POCD. Anesthetic preconditioning might be helpful for the improvement of POCD.

**Table:** 

Authors' roles & responsibilities	
SMY	Conception or design of the work; acquisition, analysis, or interpretation of data for the work; drafting the work or revising it critically for important intellectual content; final approval of the version to be published
HL	Conception or design of the work; acquisition, analysis, or interpretation of data for the work; drafting the work or revising it critically for important intellectual content; final approval of the version to be published
